# Food Insecurity Trajectories in the US During the First Year of the COVID-19 Pandemic

**DOI:** 10.5888/pcd20.220212

**Published:** 2023-01-19

**Authors:** Jin E. Kim-Mozeleski, Stephanie N. Pike Moore, Erika S. Trapl, Adam T. Perzynski, Janice Y. Tsoh, Douglas D. Gunzler

**Affiliations:** 1 Prevention Research Center for Healthy Neighborhoods, Department of Population and Quantitative Health Sciences, Case Western Reserve University, Cleveland, Ohio; 2 Center for Health Care Research and Policy, Case Western Reserve University at MetroHealth Medical Center, Cleveland, Ohio; 3 Department of Psychiatry and Behavioral Sciences, University of California, San Francisco

## Abstract

**Introduction:**

The objective of this study was to characterize population-level trajectories in the probability of food insecurity in the US during the first year of the COVID-19 pandemic and to examine sociodemographic correlates associated with identified trajectories.

**Methods:**

We analyzed data from the Understanding America Study survey, a nationally representative panel (N = 7,944) that assessed food insecurity every 2 weeks from April 1, 2020, through March 16, 2021. We used latent class growth analysis to determine patterns (or classes) of pandemic-related food insecurity during a 1-year period.

**Results:**

We found 10 classes of trajectories of food insecurity, including 1 class of consistent food security (64.7%), 1 class of consistent food insecurity (3.4%), 5 classes of decreasing food insecurity (15.8%), 2 classes of increasing food insecurity (4.6%), and 1 class of stable but elevated food insecurity (11.6%). Relative to the class that remained food secure, other classes were younger, had a greater proportion of women, and tended to identify with a racial or ethnic minority group.

**Conclusion:**

We found heterogeneous longitudinal patterns in the development, resolution, or persistence of food insecurity during the first year of the COVID-19 pandemic. Experiences of food insecurity were highly variable across the US population, with one-third experiencing some form of food insecurity risk. Findings have implications for identifying population groups who are at increased risk of food insecurity and related health disparities beyond the first year of the pandemic.

**Summary**

**What is already known on this topic?**

Food insecurity is a social determinant of health that contributes to the burden of chronic diseases and poor mental health and disproportionately affects groups with socioeconomic disadvantage.

**What is added by this report?**

We identified trajectories in the development, resolution, or persistence of food insecurity during the first year of the COVID-19 pandemic and calculated the probability of food insecurity for each trajectory. Most (64.7%) of the US population remained food secure, but one-third of the population experienced different trajectories of food insecurity.

**What are the implications for public health practice?**

Our findings quantify the heterogeneous experiences of food insecurity across the US population, with implications for identifying groups at continued risk for food insecurity beyond the first year of the COVID-19 pandemic.

## Introduction

Food insecurity — which occurs when access to adequate food to live a healthy life is limited by a lack of money or other resources — is a major public health problem in the US that contributes substantially to the burden of chronic disease and poor mental health (1). Food insecurity results in an estimated $77 billion each year in excess health care spending (2). More than 1 in 10 US households experienced food insecurity in 2020 (3). The Healthy People 2020 goal was to reduce the national prevalence of food insecurity to 6%, and this is now the target for Healthy People 2030 (4).

Along with illness and death in the US (5) and globally, the COVID-19 pandemic caused a cascade of economic and social disruptions that increased food insecurity, particularly in its earliest months (6). Data from the US Census Household Pulse Survey showed that food insufficiency (a severe form of food insecurity) rose from 8% in March 2020 to 10% in June 2020 (7). Some households, such as Black and Hispanic or Latino households and households with children, were found to be more vulnerable than other households to food insecurity as a result of the pandemic (3,7). In addition to these national prevalence studies, other studies have estimated food insecurity to be much higher in some samples (8,9); these studies differed in sample representation (online convenience vs community-based samples), study design (cross-sectional vs longitudinal), and food insecurity measures (single vs multiple items) (3,6–9).

The growing body of evidence shows that experiences of COVID-19 pandemic–related food insecurity varied substantially across the US population. However, most epidemiologic studies cross-sectionally assessed food insecurity during a reference period (eg, the previous 12 months) and did not follow respondents over time to examine variability of experiences during that time. This is an important research gap, because most households with food insecurity tend to experience episodic rather than chronic food insecurity (10). Few studies have characterized the variability of food insecurity over time (11). Measurement of the timing of food insecurity in a more granular fashion has led to important public health and clinical outcomes, including increased use of emergency departments and hospitalizations for hypoglycemia at the end of the month versus earlier weeks of the month based on claims data (12).

The objective of our study was to characterize the heterogeneity in food insecurity during the first year of the COVID-19 pandemic. We identified trajectories through repeated measures data that assessed the probability food insecurity every 2 weeks in a nationally representative US sample. Characterizing the heterogeneity in food insecurity during this pivotal period is important to inform public health efforts to reduce food insecurity and its associated health harms, to monitor the impact of food insecurity–related policies, and to prioritize efforts designed for populations at greatest risk of persistent food insecurity.

## Methods

### Data source and sampling

We used publicly available and deidentified data from the Understanding America Study (UAS), a nationally representative panel study of approximately 9,500 adult respondents conducted by the Center for Economic and Social Research at the University of Southern California. Launched in 2014, UAS is an internet panel study that uses an address-based probability sampling strategy and provides internet-connected tablets to respondents to minimize the effects of the digital divide for online survey completion. Each survey of the UAS is approved by the University of Southern California human subjects committee internal review board. In March 2020, UAS launched the Understanding Coronavirus in America tracking survey, a longitudinal series of surveys that assessed economic and health-related indicators during the COVID-19 pandemic. Surveys were administered every 2 weeks from March 10, 2020, through March 30, 2021, then monthly through July 20, 2021.

For this study, we analyzed data from waves 2 through 25, or April 1, 2020, through March 30, 2021, because these waves occurred during the first year of the pandemic and had consistent measures of food insecurity. A total of 8,425 unique participants responded to at least 1 survey during the study period. We excluded participants from analysis if they did not complete more than 1 survey (n = 429) or did not provide at least 2 waves of responses to the food insecurity measures (n = 52), yielding a total sample of 7,944 unique respondents. On average, these respondents completed 21.3 (SD, 4.7) waves for a total of 148,392 surveys. Of these surveys, 98.6% (n = 146,265) had responses to food insecurity measures. Participants provided informed consent before completing their first survey either electronically or on paper. More information on the survey methodology and sampling can be found at https://uasdata.usc.edu.

### Measures

**Food insecurity**. Three survey items were adapted from the US Department of Agriculture’s US Household Food Security Survey (13), designed to assess food insecurity in the previous 12 months. Understanding Coronavirus in America survey respondents were asked the following questions in reference to the past 7 days: 1) “Were you worried you would run out of food because of a lack of money or other resources?” 2) “Did you eat less than you thought you should because of a lack of money or other resources?” and 3) “Did you go without eating for a whole day because of a lack of money or other resources?” Answer choices were yes, no, or unsure. For the 3 questions, unsure accounted for 2.6%, 2.1%, and 1.4% of responses, respectively. Given prior research on social desirability bias when assessing food insecurity (14) and the small number of unsure responses, we considered yes and unsure to be affirmative responses. In total, 8.9% of surveys were affirmative for item 1, 7.9% for item 2, and 4.2% for item 3. We coded food insecurity status (binary outcome) as affirmative (yes or unsure) to any of the 3 items.

**Sociodemographic characteristics.** Sociodemographic characteristics were age, binary sex, marital status, race (White or Caucasian [hereinafter White], Black or African American, Native American/American Indian or Alaska Native, Asian/Asian American, Native Hawaiian or Other Pacific Islander, mixed), ethnicity (Hispanic or Latino, not Hispanic or Latino), educational attainment, annual household income, employment status, receipt of Supplemental Nutrition Assistance Program (SNAP) benefits, household composition (no children in household vs children in household), and US Census Bureau–designated geographic region (Northeast, Midwest, South, West). Sociodemographic factors are based on responses to participants’ first survey, collected from April 1, 2020, through March 2, 2021.

### Analysis

We used latent class growth analysis (LCGA) (15) to identify clusters of people (ie, latent classes) based on variability in their binary response of any food insecurity over time. LCGA is a longitudinal clustering technique that uses latent (unobserved) variables (16,17) and has similarities to other trajectory modeling techniques, including latent growth mixture modeling, but LCGA does not allow for within-class variation in trajectories (18). Classes identified by LCGA are not known a priori and are determined empirically, but because a substantial proportion of survey participants were observed to have no change in food insecurity status over time, we assigned them to either a class that remained food secure or a class that remained insecure throughout the study period (n = 5,317) (19).

We estimated a trajectory shape for each class, and participants (n = 2,627) were assigned to latent classes according to their posterior probabilities (20). The 1-class solution was first specified, which was then used as a comparison for solutions of increasing class size until the best solution was identified. To identify the optimal number of classes, we evaluated statistical indices and tests and parameter estimates, including likelihood ratio tests, such as the Vuong–Lo–Mendell–Rubin test and Lo–Mendell–Rubin adjusted test, entropy, Akaike information criterion (AIC), Bayesian information criterion (BIC), sample-size adjusted BIC, corrected AIC for small sample sizes, and assessment of class cell sizes (16) (Supplemental Table 1 in Appendix). We created a graph in Excel (Microsoft Corporation) of the average probability of endorsing food insecurity at each time within each cluster.

The food insecurity outcome was binary and represented any affirmative indication of food insecurity. We therefore relied on a commonly used approach for a latent variable model for a binary outcome, which was to assume that a normally distributed latent variable (food insecurity*) exists, from which participants respond affirmatively when the latent variable exceeds some threshold (16). That is, in these models, for individual *i* across time points

Food Insecurity*_i_* = Yes if τ < Food Insecurity*_i_* *

where τ is the threshold parameter.

The interpretation of the model coefficients in our LCGA followed from probit analyses. That is, within each cluster, the model coefficient for the linear slope is the change in the *z* score of a normal distribution for a 1-unit change in time. We assumed linear growth in food insecurity after evaluating descriptive plots and generalized estimating equations models of the longitudinal data in the entire sample. We used the MLR option in Mplus to estimate the model (21), which uses maximum likelihood to estimate the parameters but uses a robust sandwich-type estimator (Huber–White sandwich estimator) to calculate SEs (22). Robust approaches are resistant to errors under small violations of parametric assumptions. Our model also effectively handled ignorable missing data dependent on the data in hand (ie, following a missing-at-random assumption) via full information maximum likelihood. As a result, participants with missing data could still be included in the trajectory analysis for unbiased inference. In total, 80.0% of surveys were completed by all participants with responses to food insecurity questions. We examined sociodemographic factors across each trajectory for descriptive purposes in post hoc analyses.

We defined α = .05 for our level of significance in all statistical tests, which were 2-tailed. We conducted LCGA in Mplus version 8.6 (21). We used R program in the R studio environment (R Foundation for Statistical Computing) for data management and graphic displays, and we used the MplusAutomation package to automate LCGA estimation and interpretation (23). Sample weights were not included in the LCGA but were used to calculate summary statistics and summarize sociodemographic characteristics, which were calculated by using SAS version 9.4 (SAS Institute Inc).

## Results

Approximately two-thirds (60.0%) of the sample at first survey were aged 18 to 50 years (Table 1). Just over half of the sample identified as female (53.9%), were married (53.4%), and were currently working (57.2%). The proportion of self-identified race and ethnicity in this sample generally mirrored the overall US population; for race, 75.9% identified as White only, 13.0% as Black only, and 5.6% as Asian only. For ethnicity, 19.1% identified as Hispanic/Latino. Approximately one-third had a bachelor’s degree or more (33.8%), earned more than $75,000 annually (34.4%), had children in the household (34.7%), and were living in the South (38.8%).

**Table 1:** Baseline Sociodemographic Characteristics of Study Sample (N = 7,944 Respondents) at First Interview, Understanding America Study, April 2020-March 2021

Characteristic	Weighted % (95% CI)^a^
**Age group, y**	
18–35	30.6 (29.1–32.1)
36–50	29.4 (28.0–30.8)
51–65	24.4 (23.2–25.7)
>65	15.6 (14.6–16.6)
**Sex**	
Female	53.9 (52.4–55.4)
Male	46.1 (44.6–47.6)
**Marital status**	
Married	53.4 (51.9–54.9)
Separated/divorced/widowed	19.0 (17.9–20.2)
Single	27.6 (26.1–29.0)
**Race**	
American Indian or Alaska Native only	0.9 (0.7–1.2)
Asian only	5.6 (4.9–6.4)
Black only	13.0 (11.9–14.1)
Hawaiian or Pacific Islander only	0.3 (0.2–0.4)
White only	75.9 (74.6–77.3)
Multiracial	4.3 (3.6–4.9)
**Ethnicity**	
Not Hispanic or Latino	80.9 (79.5–82.4)
Hispanic or Latino	19.1 (17.6–20.5)
**Education**	
Less than high school graduate	8.8 (7.9–9.8)
High school graduate or equivalent	29.1 (27.6–30.5)
Some college/associate degree/technical school	28.3 (27.0–29.7)
Bachelor’s degree or more	33.8 (32.4–35.2)
**Annual household income, $**	
<10,000	9.0 (8.1–10.0)
10,000–24,999	14.2 (13.1–15.3)
25,000–49,999	23.1 (21.8–24.4)
50,000–74,999	19.3 (18.1–20.5)
≥75,000	34.4 (33.0–35.8)
**Employment**	
Currently working	57.2 (55.7–58.7)
Not working	9.1 (8.2–10.1)
Retired/disabled	20.7 (19.5–21.8)
Other/mixed employment	13.0 (12.0–14.1)
**Supplemental Nutrition Assistance Program recipient**	
No	84.4 (83.2–85.5)
Yes	15.6 (14.5–16.8)
**Household composition**	
No children in household	65.3 (63.9–66.8)
Children in household	34.7 (33.2–36.1)
**US region**	
Northeast	17.2 (16.0–18.4)
Midwest	20.3 (19.2–21.5)
South	38.8 (37.3–40.4)
West	23.6 (22.4–24.8)

a Weighted to the US general population. Sample weights were calculated for all respondents, first using base weights to correct for unequal probabilities of sampling and second using poststratification weights to align with US population sociodemographic characteristics.

### Food insecurity trajectories

**Figure 1:**
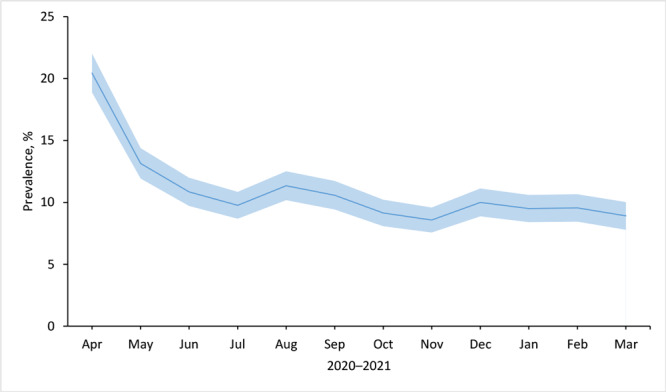
Prevalence of any food insecurity among respondents (N = 7,944) to the Understanding America Study, Understanding Coronavirus in America tracking survey, April 2020 through March 2021. Shading indicates 95% CIs.

In the early stages of the COVID-19 pandemic, in April 2020, the prevalence of any food insecurity was as high as 20.4%. The prevalence decreased to 8.9% by March 2021, with most of the decline occurring in the early months, before leveling off in mid-2020 (Figure 1). 

**Table:** 

2020, % (95% CI)									2021, % (95% CI)		
Apr	May	Jun	Jul	Aug	Sep	Oct	Nov	Dec	Jan	Feb	Mar
20.4 (18.9-22.0)	13.1 (11.9-14.4)	10.8 (9.7-12.0)	9.8 (8.7-10.8)	11.3 (10.2-12.5)	10.6 (9.4-11.7)	9.1 (8.1-10.2)	8.6 (7.6-9.6)	10.0 (8.9-11.1)	9.5 (8.4-10.6)	9.5 (8.4-10.7)	8.9 (7.8-10.0)

**Figure 2:**
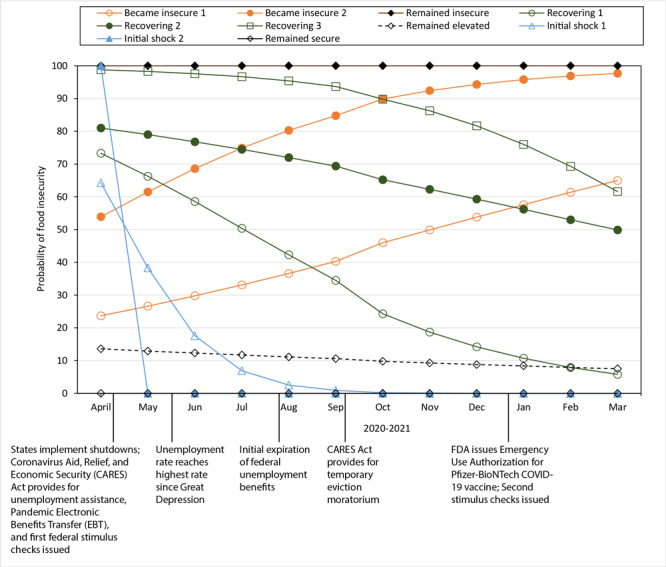
Probability of experiencing food insecurity among respondents (N = 7,944) to the Understanding America Study, Understanding Coronavirus in America tracking survey, April 2020 through March 2021. Three categories (remained secure, remained insecure, and remained elevated) were characterized as consistent. Two categories (initial shock 1 and initial shock 2) were characterized by a rapid decline in food insecurity status between March and July 2020. Three categories (recovering 1, recovering 2, and recovering 3) were defined as having experienced food insecurity initially, with the likelihood steadily declining during the first year of the COVID-19 pandemic. Two categories (became insecure 1 and became insecure 2) were defined as steadily becoming food insecure during the first year of the COVID-19 pandemic. Abbreviation: FDA, Food and Drug Administration.

We identified 10 unique trajectories of food insecurity (Figure 2), including the 2 trajectories that were empirically assigned (remained food secure and remained food insecure). Each trajectory can be described by the shape of its curve: 3 trajectories showed little change (remained insecure, remained elevated, and remained secure), 2 trajectories indicated a high probability of initial food insecurity followed by a precipitous decline (named “initial shock 1” and “initial shock 2”), 3 trajectories with a high probability of initial food insecurity followed by a steady state of decline (named “recovering 1,” “recovering 2,” and “recovering 3”), and 2 trajectories with a relatively low probability of initial food insecurity followed by a steady state of incline (named “became insecure 1” and “became insecure 2”).

**Table:** 

Trajectory	2020									2021		
	April	May	Jun	Jul	Aug	Sep	Oct	Nov	Dec	Jan	Feb	Mar
Remained secure	0	0	0	0	0	0	0	0	0	0	0	0
Initial shock 1	64.3	38.3	17.6	6.9	2.5	0.9	0.2	0.1	0	0	0	0
Initial shock 2	100	0	0	0	0	0	0	0	0	0	0	0
Remained elevated	13.6	12.9	12.3	11.7	11.1	10.6	9.8	9.3	8.8	8.4	7.9	7.5
Recovering 1	73.3	66.3	58.6	50.4	42.3	34.5	24.3	18.7	14.2	10.7	7.9	5.8
Recovering 2	81.0	79.0	76.8	74.5	72.0	69.4	65.2	62.3	59.3	56.2	53.0	49.9
Recovering 3	98.8	98.3	97.6	96.7	95.4	93.7	89.8	86.3	81.7	76.0	69.3	61.6
Remained insecure	100	100	100	100	100	100	100	100	100	100	100	100
Became insecure 1	23.7	26.6	29.8	33.1	36.6	40.3	46.0	49.9	53.8	57.6	61.4	65.0
Became insecure 2	53.9	61.5	68.6	74.9	80.3	84.8	89.9	92.4	94.3	95.8	96.9	97.7

Although most (64.7%) of the US-representative sample was in the “remained secure” category, 35.3% experienced some form of food insecurity (with 3.4% remaining insecure) during the first year of the pandemic (Table 2). Within that 35.3%, we found much variability. In addition to the 2 groups whose food security/insecurity status did not change, another group showed minimal changes: this group was mostly food secure during the study period but had an elevated prevalence of food insecurity, beginning at 13.6% in April 2020 and ending at 7.5% in March 2021. This group, “remained elevated,” comprised 11.6% of the study population.

**Table 2:** Food Insecurity Trajectories During the COVID-19 Pandemic in Study Sample (N = 7,944 Respondents), Understanding America Study, April 2020-March 2021

Trajectory	Weighted % (95% CI)^a^
**Consistent^b^**	
Remained secure	64.7 (63.2–66.2)
Remained elevated	11.6 (10.6–12.6)
Remained insecure	3.4 (2.8–4.0)
**Initial shock^c^**	
Initial shock 1	4.6 (3.9–5.3)
Initial shock 2	3.2 (2.6–3.7)
**Recovery^d^**	
Recovering 1	4.3 (3.6–4.9)
Recovering 2	2.6 (2.0–3.1)
Recovering 3	1.1 (0.8–1.5)
**Became insecure^e^**	
Became insecure 1	3.1 (2.5–3.7)
Became insecure 2	1.5 (1.1–1.9)

a Weighted to the US general population. Sample weights were calculated for all respondents, first using base weights to correct for unequal probabilities of sampling and second using poststratification weights to align with US population sociodemographic characteristics.

b Defined as minimal, if any, observed change in food security status.

c Characterized by a rapid decline in food insecurity status between March and July 2020, which may be related to initial stay-at-home or shut down orders implemented by many states and local jurisdictions or to the rapid rise in infections as well as other factors at the onset of the COVID-19 pandemic.

d Defined as having experienced food insecurity initially, but likelihood steadily declined during the first year of the COVID-19 pandemic.

e Defined as steadily becoming food insecure during the first year of the COVID-19 pandemic.

Five groups experienced some early form of food insecurity, but their probability of being food insecure declined over time. Two such trajectories, initial shock 1 (4.6% of the study population) and initial shock 2 (3.2% of the study population), appear to have briefly experienced food insecurity. This experience may have been largely related to the initial economic shutdowns in the earliest months of the pandemic in 2020, wherein states and municipalities issued stay-at-home or lockdown orders to mitigate the spread of the virus. These 2 trajectories are distinguished by the length of time needed for food insecurity to be resolved. Participants classified as initial shock 1 took about 16 weeks to resolve, while initial shock 2 was resolved within 8 weeks.

Three groups took longer to recover from food insecurity experienced earlier in the pandemic: “recovering 1” (4.3%) ended the first year at a slightly elevated probability of food insecurity, while “recovering 2” (2.6%) and “recovering 3” (1.1%) had a similar rate or slope of recovery but a different initial probability of food insecurity.

The probability of becoming food insecure increased during the first year of the pandemic for 2 groups. These 2 trajectories, “became insecure 1” (3.1%) and “became insecure 2” (1.5%), had nearly the same rate or slope of becoming food insecure but their initial probability of food insecurity differed.

Although small sample sizes and corresponding wide 95% CIs in some groups precluded a statistical comparison of sociodemographic characteristics across trajectories, characteristics appeared to differ in several ways (Supplemental Table 2A and 2B in Appendix). Those who remained food secure, compared with those in other trajectories, tended to be older, self-identified as White, and had a greater income at first interview.

## Discussion

This study is among the first to describe population-level longitudinal patterns in the development, resolution, or persistence of food insecurity during a key time of the COVID-19 pandemic, revealing heterogeneity in food insecurity experiences during this period. Notably, one-third of the population was found to experience any food insecurity when assessed longitudinally, whether food insecurity existed from the start and persisted (for approximately 15%), improved (for approximately 16%), or worsened (for approximately 5%). With careful attention to not overextend the findings of this initial study, we also observed that certain patterns of food insecurity corresponded with pivotal periods during the first year of the pandemic, starting with abrupt and widespread economic shutdowns in the earliest months. We found 2 patterns of food insecurity that occurred mainly in the earliest months of the pandemic before resolving relatively shortly thereafter (ie, initial shock 1 and initial shock 2). Therefore, an important consideration raised by our findings relates to the definition and measurement of food insecurity when accounting for the many factors that drive food insecurity.

While food insecurity at the individual level occurs when access to adequate food is limited or uncertain because of a lack of money or other resources (13), the pillars of food security in a global context also include availability, use, and stability (24). The pandemic introduced a host of other factors described elsewhere (ie, business and school closures, supply chain issues, and stockpiling of groceries and resulting shortages) (6,25) that affected experiences of food insecurity for reasons beyond a person’s lack of access caused by money. Our findings, particularly data on the initial shock 1 and initial shock 2 groups, may speak to food insecurity experiences that were driven more by societal disruptions than by economic or financial uncertainty at the individual level. For instance, while worrying about running out of food before being able to buy more is a key screening question for food insecurity (14), affirmative responses to this question could have also been driven by factors that go beyond having enough money for food. That respondents in the initial shock 2 group appeared to share similar sociodemographic characteristics with respondents in the group that remained food secure raises new considerations in defining and measuring food insecurity in the context of a novel global pandemic.

Several patterns of food insecurity described here, such as the one for people who remained food insecure during the entire study period, are particularly concerning. Because nearly half of those in the remained insecure group were SNAP recipients at the outset of the study period, this group may represent a small but important population for whom existing food insecurity protection policies were already not sufficient or whose food insecurity was not being adequately addressed before the pandemic. Groups that showed increases in the probability of food insecurity over time also warrant attention; 2 groups, “became insecure 1” and “became insecure 2,” were worse off 1 year after the onset of the pandemic than at the beginning. Although small sample sizes preclude us from making reliable sociodemographic comparisons, findings suggest that the initial probability of food insecurity distinguished the trajectories of these 2 groups.

Several lessons can be learned from populations that experienced food insecurity early in the pandemic but either recovered entirely or were headed toward recovery 1 year later. In studies of food insecurity after other large-scale and highly disruptive events, such as Hurricane Katrina in Louisiana (26) and Hurricane Harvey in Texas (27), findings highlighted several sociodemographic characteristics as protective factors (ie, income, partner status) and risk factors (ie, low levels of social support, poor physical or mental health, female sex/gender) for remaining food insecure in the year(s) after the events. Notably absent in the literature is substantive longitudinal research on the impact of recovery policies. Given the wide berth of policies implemented in the US in response to the COVID-19 pandemic, a substantial opportunity exists to explore how these policies, such as those directly aimed at reducing food insecurity or unemployment, may have contributed to each trajectory to identify what aspects of each policy either contributed to recovery or inhibited it.

A wealth of research demonstrates the relationship among income, employment status, and food insecurity. However, the confluence of various local, state, and federal responses to the pandemic provides a unique microcosm for examining a more direct impact on food insecurity. One multisite analysis found that food insecurity was highest during the pandemic among those who experienced job disruptions (28). The initial expiration of unemployment insurance at the federal level at the end of July 2020, along with the general uncertainty about its extension or even the future COVID-19 landscape, likely played a role in the upward trend in overall food insecurity observed around August 2020 (29,30). Further work in this area may entail examining how the economic impact of business closures and unemployment insurance claims (31) may have affected trajectories of food insecurity. For instance, researchers could examine the association of food insecurity trajectories with the comparative efficiency with which states implemented unemployment policies. Furthermore, research using other data sources would be necessary to examine the influence of policies such as the expanded child tax credit.

### Limitations

Our study has several limitations. Measures of food insecurity were examined only during the first year of the pandemic and did not account for food insecurity experienced before the pandemic nor their trajectory as the pandemic extended beyond March 2021. This study also assumed that all LCGA model assumptions were met in this sample for valid inference (ie, within-class multivariate normality) (32). We did perform more robust inference in case model parametric assumptions that were violated. Another limitation is that in our chosen 10-class solution, some classes had small cell sizes, and the entropy values and some of the averages of posterior probability of membership were low (2 clusters had an average posterior probability of membership below the threshold of 0.70 considered as a general guideline for adequate assignment accuracy) (33). Thus, we chose as our selection criteria global model fit and thorough interpretability of the possible clustering in this data (ie, more clusters, each unique in characteristic) over a more precise solution involving fewer classes. The small sample sizes in some classes preclude formal comparisons of sociodemographic differences across classes, although we note that those who remained food secure tended to belong to historically advantaged sociodemographic groups, whereas racial and ethnic minority populations were more likely to experience other trajectories of food insecurity (34). Across the 10 classes that were identified here, the group least vulnerable to food insecurity — remained secure — had the highest proportion of respondents who self-identified as White, earned an annual income exceeding $75,000 per year, and had completed a bachelor’s degree or more.

### Conclusion

Our findings highlighted population-level heterogeneity in the experience of food insecurity in the US throughout the first year of the pandemic, particularly in demonstrating the large variability of population response to the pandemic in food insecurity experiences. These initial findings have implications for identifying population groups who are at increased risk of food insecurity and food-insecurity–related health disparities beyond the first year of the pandemic, with particular attention to those whose risk remained high, remained elevated, or increased over time. The findings here also provide an opportunity and suggest a need for public health researchers to take a more granular look at how policies may have affected food insecurity during the first year of the pandemic.

## Appendix Supplemental Tables

**Appendix:** Supplemental Table 1. Fit Indices for Latent Class Growth Analysis for Food Insecurity Trajectories, Understanding Coronavirus in America Tracking Survey, April 2020-March 2021^a^

No. of Classes	AIC	BIC	AIC-corrected for sample size	Sample size-adjusted BIC	Entropy	Vuong–Lo–Mendell–Rubin likelihood test, *P* value	Lo–Mendell–Rubin likelihood test, *P* value
2	38,103	38,131	38,103	38,116	0.89	<.001	<.001
3	37,046	37,092	37,046	37,067	0.81	<.001	<.001
4	36,344	36,407	36,344	36,372	0.76	<.001	<.001
5	35,776	35,856	35,812	35,812	0.76	<.001	<.001
6	35,638	35,736	35,638	35,682	0.76	.003	.004
7	35,509	35,624	35,510	35,561	0.74	.002	.002
8	35,452	35,584	35,452	35,511	0.73	<.001	<.001
9	35,419	35,568	35,419	35,485	0.68	.48	.48
10	35,360	35,526	35,361	35,434	0.68	.32	.33

Abbreviations: AIC, Akaike information criterion; BIC, Bayesian information criterion.

^a^ Analysis excluded participants whose responses did not change during the study period and were grouped a priori. Study n = 2,627. We pre-fixed participants that remained food secure or food insecure into groups before employing latent class growth analysis (LCGA). LCGA was conducted on the remaining subsample with variation in their endorsement of food insecurity over time. Therefore, the 10 classes in total include the 2 pre-fixed groups, and the results of the LCGA showed that 8 classes provided the optimal solution.

**Appendix:** Supplemental Table 2A. Baseline Sociodemographic Characteristics of Participants, by Food Insecurity Trajectory, Understanding Coronavirus in America Tracking Survey, April 2020–March 2021

Characteristic	Remained insecure (n = 225)	Remained elevated (n = 834)	Remained secure (n = 5,410)	Became insecure 1 (n = 209)	Became insecure 2 (n = 102)
**Age group, y**					
18–35	44.9 (35.6–54.1)	38.3 (33.6–42.9)	26.2 (24.5–28.0)	52.9 (43.6–62.2)	38.4 (25.1–51.7)
36–50	38.9 (29.7–48.0)	28.3 (24.1–32.4)	27.6 (25.9–29.2)	30.1 (21.6–38.6)	35.7 (22.6–48.7)
51–65	12.1 (7.1–17.2)	25.6 (21.7–29.5)	26.2 (24.7–27.8)	14.4 (8.5–20.2)	18.4 (9.2–27.6)
>65	4.1 (1.0–7.3)	7.9 (5.6–10.1)	19.9 (18.6–21.3)	2.7 (0.6–4.8)	7.5 (0.8–14.3)
**Sex**					
Female	58.4 (49.1–67.6)	61.0 (56.4–65.6)	50.0 (48.2–51.8)	64.1 (55.1–73.1)	58.4 (45.1–71.6)
Male	41.6 (32.4–50.9)	39.0 (34.4–43.6)	50.0 (48.2–51.8)	35.9 (26.9–44.9)	41.6 (28.4–54.9)
**Marital status**					
Married	34.1 (25.1–43.0)	46.3 (41.6–50.9)	60.3 (58.5–62.1)	34.1 (25.3–42.9)	39.0 (25.6–52.3)
Separated/divorced/widowed	22.6 (15.1–30.0)	21.1 (17.4–24.8)	18.0 (16.7–19.4)	14.1 (8.5–19.7)	17.1 (7.5–26.6)
Single	43.4 (34.2–52.6)	32.6 (28.2–37.1)	21.7 (20.1–23.3)	51.8 (42.5–61.2)	44.0 (30.7–57.2)
**Race**					
American Indian or Alaska Native only	1.4 (0–2.8)	1.5 (0.4–2.6)	0.5 (0.4–0.7)	1.4 (0–2.9)	1.7 (0–4.0)
Asian Only	5.3 (1.0–9.5)	6.6 (4.1–9.1)	5.5 (4.6–6.4)	3.7 (0.2–7.3)	5.3 (0–11.1)
Black only	20.3 (12.5–28.1)	17.1 (13.4–20.8)	9.7 (8.5–10.9)	13.5 (7.4–19.6)	27.5 (15.2–39.8)
Hawaiian or Pacific Islander only	0.4 (0–0.9)	0.3 (0–0.6)	0.1 (0–0.1)	1.7 (0–4.2)	1.9 (0–4.7)
White only	70.2 (61.7–78.7)	68.8 (64.3–73.2)	80.0 (78.4–81.6)	72.1 (63.7–80.4)	57.4 (43.9–70.9)
Multiracial	2.5 (0.8–4.3)	5.8 (3.4–8.1)	4.2 (3.4–5.0)	7.6 (2.1–13.1)	6.3 (0–13.9)
**Ethnicity**					
Not Hispanic or Latino	62.8 (53.5–72.2)	77.1 (72.7–81.6)	84.4 (82.7–86.1)	74.3 (65.3–83.4)	77.4 (65.5–89.3)
Hispanic or Latino	37.2 (27.8–46.5)	22.9 (18.4–27.3)	15.6 (13.9–17.3)	25.7 (16.6–34.7)	22.6 (10.7–34.5)
**Education**					
Less than high school graduate	24.3 (16.4–32.2)	12.7 (9.5–15.9)	5.2 (4.3–6.1)	11.5 (5.3–17.7)	26.7 (14.5–39.0)
High school graduate or equivalent	30.3 (21.6–39.0)	30.1 (25.6–34.5)	26.1 (24.3–27.8)	39.8 (30.3–49.2)	32.1 (19.4–44.9)
Some college/associate degree/technical school	37.5 (28.5–46.5)	32.4 (28.1–36.6)	27.1 (25.5–28.6)	32.9 (24.4–41.3)	24.8 (13.8–35.9)
Bachelor’s degree or more	7.9 (3.3–12.4)	24.9 (20.9–28.9)	41.6 (39.9–43.4)	15.9 (9.4–22.3)	16.3 (6.7–25.8)
**Annual household income, $**					
<10,000	25.7 (17.5–33.9)	11.4 (8.2–14.5)	3.7 (2.9–4.5)	20.2 (12.4–28.0)	34.9 (21.9–47.9)
10,000–24,999	34.9 (26.2–43.6)	17.9 (14.4–21.5)	10.0 (8.9–11.1)	24.5 (16.6–32.4)	23.9 (13.1–34.6)
25,000–49,999	30.6 (22.0–39.2)	32.7 (28.3–37.1)	20.3 (18.8–21.8)	25.5 (17.4–33.7)	24.7 (13.0–36.4)
50,000–74,999	6.9 (2.4–11.5)	18.5 (14.9–22.1)	21.2 (19.7–22.7)	15.0 (8.5–21.5)	7.9 (0.4–15.3)
≥75,000	1.8 (0–4.4)	19.5 (15.8–23.1)	44.8 (43.0–46.7)	14.7 (7.9–21.6)	8.6 (1.1–16.2)
**Employment**					
Currently working	46.5 (37.2–55.9)	62.5 (58.1–67.0)	60.6 (58.8–62.4)	52.0 (42.6–61.4)	39.9 (26.7–53.2)
Not working	22.2 (14.4–30.0)	10.1 (7.3–13.0)	5.4 (4.5–6.4)	18.5 (10.9–26.1)	19.8 (9.7–30.0)
Retired/disabled	18.8 (12.1–25.5)	14.0 (11.1–16.9)	22.7 (21.3–24.1)	11.2 (5.6–16.7)	21.1 (10.4–31.8)
Other/mixed employment	12.5 (6.8–18.1)	13.3 (10.2–16.4)	11.3 (10.1–12.4)	18.3 (10.8–25.8)	19.1 (8.1–30.1)
**SNAP recipient**					
No	54.5 (45.2–63.7)	80.2 (76.4–84.0)	91.6 (90.5–92.7)	67.4 (58.4–76.5)	53.3 (39.9–66.8)
Yes	45.5 (36.3–54.8)	19.8 (16.0–23.6)	8.4 (7.3–9.5)	32.6 (23.5–41.6)	46.7 (33.2–60.1)
**Household composition**					
No children in household	67.1 (58.3–75.9)	58.6 (53.9–63.2)	67.2 (65.4–69.0)	61.9 (52.9–71.0)	56.2 (42.9–69.5)
Children in household	32.9 (24.1–41.7)	41.4 (36.8–46.1)	32.8 (31.0–34.6)	38.1 (29.0–47.1)	43.8 (30.5–57.1)
**US region**					
Northeast	8.3 (3.0–13.6)	15.1 (11.6–18.6)	18.5 (17.0–20.0)	19.8 (11.8–27.8)	13.7 (4.4–22.9)
Midwest	17.1 (9.9–24.3)	18.3 (14.9–21.7)	21.7 (20.3–23.1)	18.4 (11.6–25.2)	20.3 (10.0–30.6)
South	54.6 (45.4–63.8)	42.6 (38.0–47.3)	36.1 (34.3–37.9)	37.3 (27.9–46.6)	46.6 (33.0–60.1)
West	20.0 (13.5–26.4)	23.9 (20.2–27.7)	23.7 (22.2–25.2)	24.6 (17.2–31.9)	19.5 (10.6–28.3)

Abbreviation: SNAP, Supplemental Nutrition Assistance Program.

**Appendix:** Supplemental Table 2B. Baseline Sociodemographic Characteristics of Participants, by Food Insecurity Trajectory, Understanding Coronavirus in America Tracking Survey, April 2020-March 2021

Characteristic	Initial shock 1 (n = 328)	Initial shock 2 (n = 273)	Recovering 1 (n = 326)	Recovering 2 (n = 151)	Recovering 3 (n = 86)
**Age group, y**					
18–35	28.9 (22.1–35.8)	27.7 (20.0–35.3)	39.9 (32.1–47.7)	41.8 (31.3–52.3)	38.8 (23.2–54.4)
36–50	32.7 (25.5–39.8)	42.1 (33.7–50.6)	31.3 (23.7–39.0)	35.5 (25.3–45.7)	37.2 (22.3–52.2)
51–65	25.4 (18.9–31.9)	19.9 (13.7–26.0)	20.2 (14.4–25.9)	19.6 (11.3–27.8)	19.0 (7.8–30.1)
>65	13.0 (8.1–18.0)	10.3 (5.6–15.0)	8.6 (4.4–12.9)	3.1 (0–7.0)	5.0 (0–10.5)
**Sex**					
Female	54.3 (46.8–61.9)	49.5 (41.1–58.0)	70.3 (62.8–77.8)	70.3 (60.7–79.8)	67.0 (51.8–82.2)
Male	45.7 (38.1–53.2)	50.5 (42.0–58.9)	29.7 (22.2–37.2)	29.7 (20.2–39.3)	33.0 (17.8–48.2)
**Marital status**					
Married	42.9 (35.4–50.3)	50.8 (42.4–59.2)	36.0 (28.3–43.7)	34.4 (24.4–44.4)	21.8 (11.2–32.4)
Separated/divorced/widowed	22.3 (16.3–28.4)	20.6 (13.8–27.4)	23.7 (17.4–30.1)	20.5 (11.7–29.4)	21.7 (9.9–33.6)
Single	34.8 (27.5–42.1)	28.6 (20.9–36.3)	40.2 (32.4–48.1)	45.0 (34.4–55.6)	56.5 (41.7–71.3)
**Race**					
American Indian or Alaska Native only	1.4 (0.1–2.7)	0.7 (0–1.7)	4.1 (1.2–7.0)	0.8 (0–2.1)	0.8 (0–2.2)
Asian only	6.0 (2.6–9.4)	9.4 (4.0–14.8)	4.0 (1.2–6.9)	3.3 (0–6.8)	9.4 (0.4–18.3)
Black only	18.8 (12.8–24.8)	15.3 (9.1–21.5)	21.0 (14.2–27.7)	28.7 (18.9–38.5)	18.6 (6.3–31.0)
Hawaiian or Pacific Islander only	0.1 (0–0.2)	0.5 (0–1.5)	1.1 (0–2.2)	0.6 (0–1.8)	2.5 (0–5.3)
White only	70.3 (63.5–77.1)	72.1 (64.4–79.8)	66.7 (59.2–74.1)	65.1 (54.9–75.2)	63.7 (49.1–78.2)
Multiracial	3.4 (1.0–5.9)	2.0 (0–4.4)	3.2 (1.1–5.3)	1.6 (0.2–2.9)	5.0 (0–10.2)
**Ethnicity**					
Not Hispanic or Latino	78.7 (71.7–85.7)	84.5 (77.8–91.2)	66.9 (58.8–75.1)	70.2 (59.8–80.7)	74.8 (60.1–89.4)
Hispanic or Latino	21.3 (14.3–28.3)	15.5 (8.8–22.2)	33.1 (24.9–41.2)	29.8 (19.3–40.2)	25.2 (10.6–39.9)
**Education**					
Less than high school graduate	17.2 (11.0–23.4)	8.2 (3.4–12.9)	14.0 (8.3–19.8)	21.4 (12.9–29.9)	18.7 (6.5–31.0)
High school graduate or equivalent	37.0 (29.6–44.5)	28.9 (20.8–37.0)	45.5 (37.5–53.5)	37.4 (26.8–48.0)	40.4 (25.0–55.8)
Some college/associate degree/technical school	26.1 (19.7–32.5)	33.9 (26.2–41.6)	26.1 (19.8–32.4)	28.2 (18.7–37.7)	27.0 (14.0–40.0)
Bachelor’s degree or more	19.6 (14.2–25.0)	29.0 (21.5–36.5)	14.4 (9.2–19.5)	13.0 (6.5–19.5)	13.9 (2.9–24.8)
**Annual household income, $**					
<10,000	19.4 (13.2–25.6)	8.2 (3.8–12.6)	23.8 (16.8–30.7)	31.1 (21.1–41.0)	26.9 (13.0–40.8)
10,000–24,999	22.4 (16.1–28.7)	14.4 (8.4–20.4)	24.8 (18.4–31.2)	18.8 (11.3–26.4)	26.3 (12.7–39.9)
25,000–49,999	21.1 (15.1–27.1)	23.5 (16.4–30.6)	29.2 (22.0–36.3)	28.1 (18.4–37.8)	28.3 (14.7–41.9)
50,000–74,999	15.5 (9.8–21.1)	23.8 (16.5–31.1)	14.5 (8.6–20.4)	14.9 (7.2–22.6)	14.8 (2.8–26.8)
≥75,000	21.6 (15.7–27.5)	30.1 (22.3–37.9)	7.8 (3.4–12.2)	7.1 (1.2–12.9)	3.6 (0–8.0)
**Employment**					
Currently working	40.4 (33.1–47.7)	59.5 (51.3–67.7)	42.8 (34.8–50.7)	34.9 (24.9–45.0)	41.6 (26.0–57.3)
Not working	18.4 (12.2–24.6)	9.2 (4.4–14.0)	21.1 (14.4–27.8)	23.4 (13.9–32.8)	13.7 (3.9–23.4)
Retired/disabled	22.2 (16.1–28.2)	19.5 (13.1–25.9)	19.1 (13.4–24.7)	15.9 (8.2–23.7)	20.2 (8.4–31.9)
Other/mixed employment	19.0 (12.9–25.1)	11.8 (6.7–16.9)	17.1 (11.3–22.9)	25.8 (16.4–35.1)	24.5 (11.5–37.5)
**SNAP recipient**					
No	74.6 (67.8–81.5)	84.6 (78.6–90.6)	63.9 (56.2–71.7)	55.5 (44.7–66.3)	69.0 (54.8–83.2)
Yes	25.4 (18.5–32.2)	15.4 (9.4–21.4)	36.1 (28.3–43.8)	44.5 (33.7–55.3)	31.0 (16.8–45.2)
**Household composition**					
No children in household	66.1 (58.9–73.3)	60.8 (52.5–69.1)	67.0 (59.4–74.7)	60.2 (49.7–70.7)	61.1 (45.9–76.4)
Children in household	33.9 (26.7–41.1)	39.2 (30.9–47.5)	33.0 (25.3–40.6)	39.8 (29.3–50.3)	38.9 (23.6–54.1)
**US region**					
Northeast	16.8 (10.7–22.8)	19.1 (11.7–26.6)	9.7 (5.0–14.4)	17.8 (9.1–26.6)	8.8 (0–17.9)
Midwest	21.1 (15.0–27.2)	13.2 (8.0–18.4)	15.1 (9.8–20.4)	8.7 (3.7–13.7)	40.7 (25.2–56.3)
South	42.6 (35.1–50.2)	35.8 (27.6–44.0)	55.2 (47.5–62.9)	46.0 (35.3–56.7)	19.4 (7.3–31.5)
West	19.4 (14.5–24.3)	31.9 (24.4–39.3)	20.0 (14.9–25.1)	27.4 (18.5–36.4)	31.0 (17.4–44.6)

Abbreviation: SNAP, Supplemental Nutrition Assistance Program.
